# Association of obesity with orbital fat expansion in thyroid eye disease

**DOI:** 10.1186/s12886-024-03824-9

**Published:** 2025-01-02

**Authors:** Po-Chin Kuo, Shu-Chun Kuo, Yi-Shan Teng, Chun-Chieh Lai

**Affiliations:** 1 Department of Ophthalmology, National Cheng Kung University Hospital, College of Medicine, National Cheng Kung University, No. 138, Sheng Li Road, Tainan, 704 Taiwan; 2 Department of Ophthalmology, Chi Mei Medical Center, Tainan, Taiwan; 3 Department of Optometry, Chung Hwa University of Medical Technology, Tainan, Taiwan; 4 Department of Dermatology, Kaohsiung Medical University Hospital, Kaohsiung Medical University, Kaohsiung, Taiwan; 5 Institute of Clinical Medicine, College of Medicine, National Cheng Kung University, Tainan, Taiwan

**Keywords:** Obesity, Orbital fat, Thyroid eye disease, Proptosis, Exophthalmos

## Abstract

**Background:**

To investigate the association between obesity and orbital fat expansion in proptosis of thyroid eye disease.

**Methods:**

This observational study retrospectively enrolled 87 participants who received orbital fat decompression surgery for thyroid eye disease. Primary outcome measures included average body mass index (BMI) and the proportion of the study sample with overweight and obesity, compared with the general Taiwanese population. Secondary outcome measures included the association of obesity with proptosis severity, removed fat volume, and thyroid status.

**Results:**

The average BMI (25.59 ± 4.36 kg/m^2^) of the study sample was significantly higher than that in the general population of Taiwan (24.5 kg/m^2^; *P* = 0.012). Participants with overweight (19.52 ± 3.52 mm) and obesity (21.25 ± 3.76 mm) exhibited significantly more severe proptosis than participants without overweight (18.05 ± 3.37 mm) and without obesity (18.09 ± 3.02 mm; *P* = 0.029 and *P* < 0.001, respectively). In addition, a significantly greater orbital fat volume was removed from the group with obesity (4.61 ± 1.17 ml) versus that without obesity (3.57 ± 1.12 ml; *P* = 0.021). A positive correlation between BMI and removed fat volume was noted (correlation coefficient = 0.291, *P* = 0.005). BMI was an independent factor predicting both proptosis severity (*P* < 0.001) and removed orbital fat volume (*P* = 0.02).

**Conclusions:**

Obesity is associated with orbital fat expansion and consequently more severe proptosis in thyroid eye disease. Weight control may be a potential strategy to prevent thyroid-associated exophthalmos.

## Background

Thyroid eye disease (TED), an inflammatory autoimmune disorder that affects orbital and periorbital tissue, is characterized by unilateral or bilateral proptosis. TED affects people not only cosmetically, but also physically. Periorbital expansion causes diplopia, and furthermore, progression to dysthyroid optic neuropathy can threaten eyesight. Orbital fat proliferation plays a critical pathological role in the periorbital expansion of TED [1]. However, whether a correlation exists between obesity and orbital fat accumulation in thyroid-associated orbitopathy remains undetermined.

Studies have identified adipogenesis to be the mechanism of orbital fat deposition in TED [2, 3]. Lacheta et al. noted the capacity of orbital fibroblasts to differentiate into adipocytes [4]. Khong et al. also reported enhanced adipogenesis in thyroid ophthalmopathy, including the proliferation and differentiation of adipocytes [5]. Adipogenesis, namely the proliferation of adipocyte precursor cells and their differentiation into mature adipocytes, contributes to obesity. Therefore, we hypothesized that obesity may be associated with orbital fat expansion in TED.

Our study aims to investigate the association between obesity and proptosis in TED. We evaluated whether participants who underwent orbital fat decompression surgery had a greater average body mass index (BMI). The study also explored the association of obesity with proptosis severity and the correlation between obesity and removed fat volume from orbital fat decompression surgery.

## Methods

### Subjects

This cross-sectional, observational study retrospectively enrolled participants who received orbital fat decompression surgery between January 2015 and February 2022 in a single tertiary referral center. The study followed the tenets of the Declaration of Helsinki. Institutional Review Board approval by National Cheng Kung University Hospital was obtained, and an informed consent was waived. All participants had a diagnosis of TED and had at least one of the following surgical indications: (1) proptosis of either eye with a protrusion value over 18 mm as measured by a Hertel exophthalmometer, namely the upper limit of normal Hertel value in the Chinese population [6, 7], (2) an asymmetry between the protrusion of both eyes of 2 mm or greater [8], and (3) self-reported unsatisfactory and disfiguring exophthalmos. This study excluded patients who (1) had previously received this surgery or (2) had undergone simultaneous bone decompression surgery. In the case of bilateral fat decompression surgery, only the eye with more severe proptosis was selected, due to between-eye correlations and statistical assumptions of independence of the data [9, 10].

### Surgical technique

**Fig. 1:**
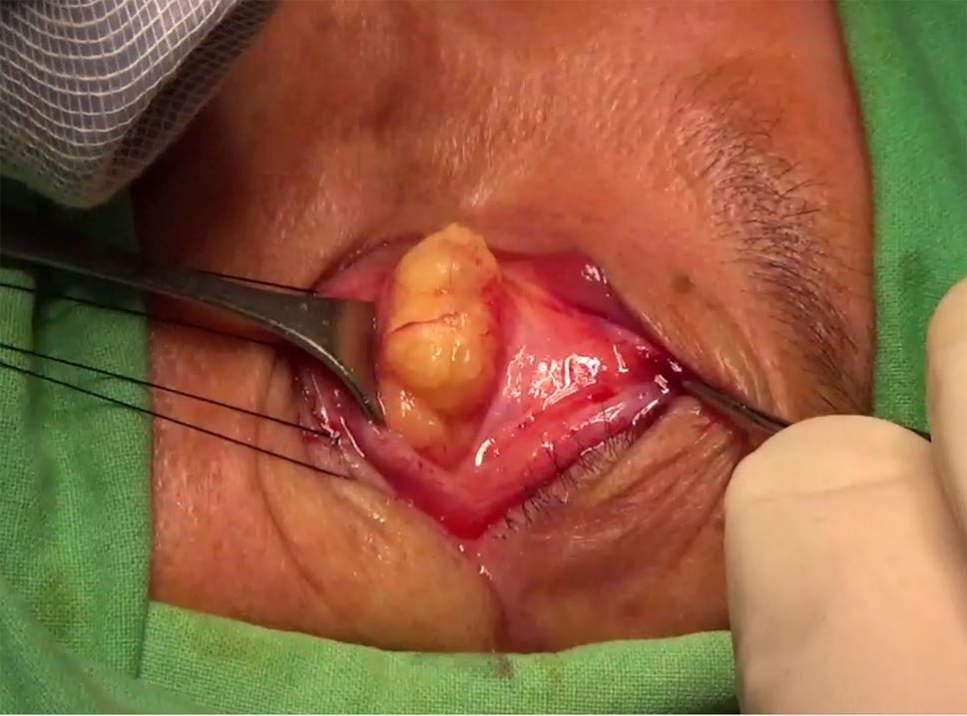
Exposure of hypertrophic fat in orbital fat decompression surgery

All enrolled participants underwent orbital fat decompression surgery by a single surgeon (CCL). An anterior orbitotomy was performed under general anesthesia with a horizontal incision made through the conjunctiva of the lower fornix [11]. Hypertrophic fat, including medial, middle, and lateral fat pads, was then removed for orbital decompression (Fig. 1). The surgical goal was to achieve proptosis reduction equal to 15 mm, namely the average Hertel exophthalmometric value in the healthy Chinese population [7], or bilateral symmetry in patients with an asymmetry of 2 mm or greater between both eyes. The desired volume of adipose tissue was calculated by the predictive equation developed by Liao et al. with a 0.8 mm Hertel change for each milliliter of orbital fat resection [12].

### Outcome measures

The primary outcome measure was participant BMI. Secondary outcome measures included the preoperative protrusion value, removed orbital fat volume during surgery, and preoperative thyroid status. Demographic data were obtained and reviewed from medical records.

BMI was measured and calculated on the day of surgery admission. BMI levels were categorized as normal (BMI ≥ 18.5 and < 24 kg/m2), overweight (BMI ≥ 24 and < 27 kg/m2), and obese (BMI ≥ 27 kg/m2) as defined by the Health Promotion Administration, Ministry of Health and Welfare, Taiwan. The reference BMI of the general Taiwanese population was documented according to the 2017 to 2020 Nutrition and Health Survey in Taiwan (NAHSIT) as administered by the Health Promotion Administration, Ministry of Health and Welfare, Taiwan. The preoperative protrusion value was measured by a Hertel exophthalmometer within 1 month prior to surgery. Removed orbital fat volume was directly measured with a syringe from the surgical specimen. Thyroid status was defined by blood thyroid-stimulating hormone (TSH) level measured within 3 months prior to surgery and was categorized into hyperthyroidism (TSH < 0.25 µU/ml), euthyroidism (TSH ≥ 0.25 and ≤ 4 µU/ml), and hypothyroidism (TSH > 4 µU/ml) [13–15].

### Statistical analysis

Statistical analyses were performed using SPSS version 17.0 (SPSS, Chicago, IL, USA). We compared the mean BMI of enrolled participants with that of the general Taiwanese population using a one-sample *t* test. Subgroup analyses of sex and age were performed. We applied a binomial test to compare the proportion of participants with overweight or obesity versus the proportion of individuals in the general Taiwanese population. We compared the proptosis severity and average fat volume removed between the groups with and without overweight and between the groups with and without obesity with a two-sample *t* test. We verified the association between BMI and fat volume removed by the Pearson correlation coefficient. We examined the contributions of different factors, including age, sex, and BMI, on proptosis severity and removed fat volume through multivariable linear regression analyses. A one-way analysis of variance was performed to compare the BMI of participants with varying thyroid statuses. Statistical significance was indicated at *P* < 0.05.

## Results

### Participant characteristics

A total of 87 participants, including 32 men (36.8%) and 55 women (63.2%), were included in this study. The average age of participants was 48.66 ± 12.67 years (range = 23 to 87 years). The average BMI was 25.59 ± 4.36 kg/m^2^. Among the participants, 30 (34.5%) had overweight (BMI ≥ 24 and < 27 kg/m^2^) and 24 (27.6%) had obesity (BMI ≥ 27 kg/m^2^). The average proptosis value measured by a Hertel exophthalmometer was 18.96 ± 3.52 mm.

### BMI analysis

The study noted a significantly greater average BMI (25.59 ± 4.36 kg/m^2^) in enrolled participants compared with the general adult Taiwanese population (24.5 kg/m^2^; *P* = 0.012; Table 1). A subgroup analysis by sex also indicated a greater average BMI among male (26.78 ± 4.16 kg/m^2^) and female (24.89 ± 4.36 kg/m^2^) participants versus their counterparts in the Taiwanese adult population (25.3 kg/m^2^ for men and 23.8 kg/m^2^ for women; *P* = 0.026 and 0.035, respectively; Table 1). The subgroup analysis by age indicated a significantly higher BMI for participants aged 19 to 44 years versus their counterparts in the Taiwanese adult population (25.96 ± 4.87 kg/m^2^; 24.3 kg/m^2^ for Taiwanese population aged 19 to 44; *P* = 0.027; Table 1).

**Table 1:** Average BMI of study sample and that of general Taiwanese population for comparison

	BMI (Mean ± SD) (kg/m^2^)		
	Study sample	Reference population^a^	*P* value
All	25.59 ± 4.36 (*n* = 87)	24.5 ± 0.12	0.012*
**Sex**			
Male	26.78 ± 4.16 (*n* = 32)	25.3 ± 0.16	0.026*
Female	24.89 ± 4.36 (*n* = 55)	23.8 ± 0.13	0.035*
**Age**			
19–44	25.96 ± 4.87 (*n* = 34)	24.3 ± 0.19	0.027*
45–64	25.35 ± 4.08 (*n* = 47)	24.7 ± 0.15	0.139
≥ 65	25.25 ± 3.97 (*n* = 6)	25.1 ± 0.09	0.465

^a^Reference population: 2017 to 2020 Nutrition and Health Survey in Taiwan, administered by the Health Promotion Administration, Ministry of Health and Welfare, Taiwan

*P < 0.05

The study sample had a significantly greater proportion (62.1%) of people with overweight and obesity (BMI ≥ 24 kg/m^2^) compared with the Taiwanese adult population (50.7%; *P* = 0.022; Table 2). The subgroup analysis by sex indicated a significantly greater proportion (75%) of men with overweight and obesity (58.8% for Taiwanese adult population; *P* = 0.043; Table 2). The study sample also had a nonsignificantly greater proportion of individuals with obesity (27.6%) than the general population in Taiwan (23.9%; *P* = 0.244; Table 3).

**Table 2:** Proportions of people with overweight and obesity in study sample and in general Taiwanese population for comparison

	Study sample		Reference population^a^	
	BMI < 24 (%)	BMI ≥ 24 (%)	BMI ≥ 24 (%)	*P* value
All	37.9 (*n* = 33)	62.1 (*n* = 54)	50.7	0.022*
**Sex**				
Male	25 (*n* = 8)	75 (*n* = 24)	58.8	0.043*
Female	45 (*n* = 25)	55 (*n* = 30)	42.8	0.053

^a^Reference population: 2017 to 2020 Nutrition and Health Survey in Taiwan, administered by the Health Promotion Administration, Ministry of Health and Welfare, Taiwan

*P < 0.05

**Table 3:** Proportions of people with obesity in study sample and in general Taiwanese population for comparison

	Study sample		Reference population^a^	
	BMI < 27 (%)	BMI ≥ 27 (%)	BMI ≥ 27 (%)	*P* value
All	72.4 (*n* = 63)	27.6 (*n* = 24)	23.9	0.244
**Sex**				
Male	59.4 (*n* = 19)	40.6 (*n* = 13)	28.3	0.091
Female	80 (*n* = 44)	20 (*n* = 11)	19.6	0.524

^a^Reference population: 2017 to 2020 Nutrition and Health Survey in Taiwan, administered by the Health Promotion Administration, Ministry of Health and Welfare, Taiwan

### Proptosis severity analysis

The group with overweight (BMI ≥ 24 kg/m^2^) had a significantly greater proptosis value (19.52 ± 3.52 mm) than that without overweight (18.05 ± 3.37 mm; *P* = 0.029; Table 4). Similarly, the group with obesity (BMI ≥ 27 kg/m^2^) had a significantly greater proptosis value (21.25 ± 3.76 mm) than that without obesity (18.09 ± 3.02 mm; *P* < 0.001; Table 4). A multivariable linear regression analysis suggested a positive correlation of BMI (beta coefficient 0.416; *P* < 0.001), and a negative correlation of age (beta coefficient − 0.295; *P* = 0.002), with proptosis severity (Table 5).

**Table 4:** Proptosis value between groups with and without overweight and between groups with and without obesity

	BMI < 24 (*n* = 33)	BMI ≥ 24 (*n* = 54)	*P* value
Proptosis^a^ (mm) (Mean ± SD)	18.05 ± 3.37	19.52 ± 3.52	0.029*
	**BMI < 27** (***n*** = **63**)	**BMI ≥ 27** (***n*** = **24**)	***P*** **value**
Proptosis^a^ (mm) (Mean ± SD)	18.09 ± 3.02	21.25 ± 3.76	< 0.001*

^a^Measured by Hertel exophthalmometer

*P < 0.05

**Table 5:** Multivariable linear regression analysis of the independent factors for proptosis severity

	*B* ^a^	*P*
Age	-0.295	0.002*
Sex	-0.180	0.055
BMI	0.416	< 0.001*

^a^Standardized beta coefficient

*P < 0.05

### Fat volume analysis

**Fig. 2:**
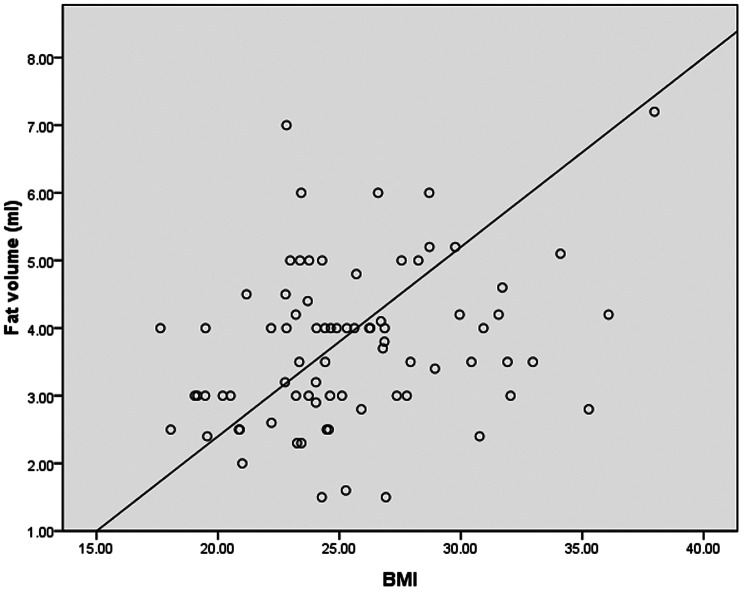
Relationship between BMI and removed orbital fat volume in orbital fat decompression surgery. Correlation coefficient = 0.291, *P* = 0.005

The study noted that a greater fat volume (3.81 ± 1.14 ml) was removed from the group with overweight (BMI ≥ 24 kg/m^2^) in orbital fat decompression surgery than from that without overweight (3.61 ± 1.19 ml; *P* = 0.24; Table 6). A significantly greater orbital fat volume (4.61 ± 1.17 ml) was removed from the group with obesity (BMI ≥ 27 kg/m^2^) than from that without obesity (3.57 ± 1.12 ml; *P* = 0.021; Table 6). A multivariable linear regression analysis predicting the removed fat volume in orbital fat decompression surgery revealed that BMI was the only factor significantly associated with removed orbital fat volume (*P* = 0.02; Table 7). A positive correlation between BMI and removed fat volume was demonstrated (correlation coefficient = 0.291, *P* = 0.005; Fig. 2).

**Table 6:** Removed orbital fat volume of groups with and without overweight, and with and without obesity

	BMI < 24 (*n* = 33)	BMI ≥ 24 (*n* = 54)	*P* value
Fat volume (ml) (Mean ± SD)	3.61 ± 1.19	3.81 ± 1.14	0.235
	BMI < 27 (*n* = 63)	BMI ≥ 27 (*n* = 24)	*P* value
Fat volume (ml) (Mean ± SD)	3.57 ± 1.12	4.16 ± 1.17	0.021*

*P < 0.05

**Table 7:** Multivariable linear regression analysis of the independent factors for removed orbital fat volume

	*B* ^a^	*P*
Age	-0.007	0.489
Sex	-0.232	0.389
BMI	0.074	0.020*

^a^Standardized beta coefficient

*P < 0.05

### Thyroid status analysis

Twenty-four study participants exhibited euthyroidism (TSH ≥ 0.25 and ≤ 4 µU/ml), 20 exhibited hyperthyroidism (TSH < 0.25 µU/ml) and 11 exhibited hypothyroidism (TSH > 4 µU/ml). The TSH levels of the remaining 32 participants were not available. A one-way analysis of variance did not detect a significant difference in average BMI among participants with different thyroid statuses (*P* = 0.449; Table 8).

**Table 8:** Average BMI of hyperthyroid, euthyroid, and hypothyroid groups

	TSH < 0.25 µU/ml (*n* = 20)	0.25 ≤ TSH ≤ 4 µU/ml (*n* = 24)	TSH > 4 µU/ml (*n* = 11)	*P* value
BMI (Mean ± SD) (kg/m^2^)	25.13 ± 4.95	26.98 ± 5.22	25.96 ± 3.50	0.449

## Discussion

The present study revealed an association between obesity and exophthalmos in TED. Participants who underwent orbital fat decompression surgery had a greater average BMI than the general population of Taiwan. Participants with obesity exhibited significantly more severe proptosis. Furthermore, significantly greater orbital fat volume was obtained from the group with obesity in orbital fat decompression surgery.

Participants who received orbital fat decompression surgery had a significantly greater average BMI than the general population of Taiwan. We also observed a greater proportion of overweight and obesity in the study sample. Adipogenesis is the process during which preadipocytes mature into adipocytes, which results in fat expansion. When whole-body metabolic homeostasis is altered, excess adipose tissue deposits result in the development of obesity [16]. Crisp et al. and previous studies have noted the potential role of orbital adipocytes in the increased orbital volume of TED [3]. Lu et al. also proved the association of obesity-related factors, including BMI, with Graves’ orbitopathy [17]. Therefore, we hypothesized that participants with obesity may experience more active adipogenesis and have a greater tendency toward fat deposition, and thus experience more severe proptosis in TED. Notably, the age subgroup analysis indicated a significantly greater average BMI in the study sample among individuals aged 19 to 44 relative to their counterparts in the general population, which agrees with findings in the literature. The enlargement of the extraocular muscles predominates among older patients; by contrast, fatty hypertrophy predominates among younger patients [18]. Ugradar et al. also observed a negative correlation between orbital fat volume and age [19]. The difference in predominant type by age may result from the diminishing adipogenic potential of orbital fibroblasts with aging [18, 20].

The study revealed an association of proptosis severity with overweight and obesity. Participants with overweight and obesity tended to have greater proptosis values. Proptosis severity is reported to be closely related to orbital adipose tissue volume [21, 22]. We therefore hypothesized that participants with a greater BMI, and consequently with a higher potential for orbital fat deposition, would develop more severe proptosis. With a multivariable linear regression analysis, the study also demonstrated that BMI was an independent factor positively correlated with proptosis severity. Although age was also found to be a significant factor negatively correlated with proptosis severity, we inferred that this was due to adipose tissue atrophy and fat volume loss along with aging. Additionally, age-related orbital fat herniation anteriorly through the infraorbital space due to weakening of supportive tissue, including orbital rim bony resorption, loosened capsulopalpebral fascia, and loss of muscle tone, also makes proptosis less prominent in the elderly. Instead of intraorbital deposition, which can lead to proptosis, orbital fat in the elderly herniates anteriorly, resulting in baggy eyelids. To investigate whether orbital fat volume contributed to the greater proptosis value in the group with obesity, the study established a positive correlation between BMI and removed fat volume in orbital fat decompression surgery. A significantly greater fat volume was removed in the group with obesity. The technique of orbital fat decompression surgery effectively reduced the degree of proptosis in TED through fat removal [1]. The activation of further adipogenesis in participants with obesity made a greater volume of fat available for manipulation and removal during orbital fat decompression surgery. To sum up, when taking both proptosis severity and removed orbital fat volume into account, BMI has been the only, common, significant factor in the study.

A significant association between thyroid status and BMI was absent from this present study. However, studies have observed lower BMI and blood lipid levels in subclinical hyperthyroidism [23, 24]. We attribute the results to several factors. Due to the retrospective design of this study, data on the thyroid status of more than one-third of the participants was missing, and the analysis was therefore limited by the relatively small sample size. Furthermore, most participants were currently under or had received medical treatment for dysthyroidism; antithyroid drugs can interfere with measurement reliability.

The present study revealed the association between obesity and exophthalmos in TED from multiple aspects; however, it has several limitations. First, although a correlation was indicated between obesity and proptosis in TED, the cause-and-effect relationship remains undetermined. Moreover, surgical specimen measurements were used instead of radiological measurements of orbital fat volume. This method potentially led to an underestimation of orbital fat volume, particularly when the ophthalmologist encountered technical difficulties during fatty decompression.

## Conclusions

In conclusion, our study demonstrated an association between obesity and orbital fat expansion and proptosis in TED. Our research reveals that because orbital fat deposition in thyroid-associated orbitopathy is correlated with obesity, weight control is a potentially crucial strategy to prevent patients with thyroid orbitopathy from developing severe exophthalmos. We propose an emphasis on weight control in routine care for patients with thyroid disorders. Future prospective studies are required to establish the effect of body weight reduction or physical activity on TED activity.

## Data Availability

The datasets used and/or analyzed during the current study are available from the corresponding author on reasonable request.

## Abbreviations

TED: Thyroid eye disease
BMI: Body mass index
